# The Matrons' Experiences

**Published:** 1916-04-29

**Authors:** 


### THE MATRONS' AND SISTERS' DEPARTMENT.

### THE MATRONS' EXPERIENCES.

The College of Nursing being now well on its way, an opportunity is created for many workers in the hospital field to do their part to help it forward and make it a splendid success. Each matron, in proportion to the number of her working years and her varied experience, might help the work by jotting down, after due thought, the chief aids, hindrances, and difficulties she has encountered in the selection, education, training, and employment of nurses, not only in hospitals and kindred institutions, but in branches of the nursing field of which she may have intimate knowledge. The College of Nursing should, at any rate, facilitate a fuller interchange of ideas, mutual co-operation in cases of doubt or difficulty, a wider outlook, and a more accurate and complete knowledge. This should be followed by a resulting progressive development not only for the leaders, but for the best minds and most zealous workers the profession contains.

The following notes have been spontaneously sent by " a Matron of a Provincial General Hospital not far from the coast ":

Here are her jottings: " These dear wounded and sick men from the Front! Happy is the hospital that has been allowed to open some of its wards to receive them, and so take its part in this great world struggle. A most honourable privilege is that of the honorary staff and residents who all gladly accept the burden, for the additional work is heavy. Some older nurses, many years working in the groove some call the 'hospital groove,' began by thinking it would be impossible to get more into a day's work, are now happily surprised it is possible to double it.

" What a new thrill is experienced when the Red Cross motors draw up to the open hall door and the V.A.D. and St. John's men unload the burdens and a convoy of sixteen, eighteen, twenty-five, or more dear patient men, labelled as if they were bales of goods, are laid on the hall floor one at a time, none complaining; and then the wards, so warm and bright! Warm, clean shirts, hot tea, cake or bread and butter, and perhaps smokes. Glad of this welcome—so true and eager—many a hospital has been illuminated by the charge of warrior patients.

" The cheerful life these soldiers make—many so young!—and even those dreadful gramophones and chorus songs are tolerated; and presently the garden will be full of warriors in their blue suits, making colour amongst the green. Such nice lads, running round for postcards to let their friends know they are safe in England. And a mixture; one young private owns more than one motor-car, the private in the next bed hasn't a penny for a smoke, yet the best of comrades all. There is a sad note at times when one of them has to hand in his discharge to the Great Paymaster, but what a tender earthly organisation is that which sends the poor remains free of cost to be laid at rest perhaps 200 miles away! "Who would economise in this matter? The coming months may see heavier convoys arrive. Let us all, General Hospitals, Territorials, V.A.D., and Military Hospitals, be grateful for the past and ready for the future. The trained and training nurses welcoming gladly the hard-working V.A.D. nurse, whose willing spirit may perhaps help to rekindle some of the old enthusiasm and love, monotonous work had somewhat chilled."

We have given the matron's own words, hoping others will write. For if the outside world in each locality can, through the matron's co-operation with The Hospital, be made to realise our voluntary hospitals in war-time as they are in fact, the results may be fruitful for good in a variety of ways—not only to hospital workers and their patients, but to the outside public who, to their credit, are full of desire to help those who serve, by encouragement, by cash, by personal service, and by every means within their individual compass.

